# Postpartum Depression: Overlap Between Symptom‐Based and Register‐Based Measures

**DOI:** 10.1111/acps.70090

**Published:** 2026-04-14

**Authors:** Sofie Egsgaard, Mette Bliddal, Mette‐Marie Zacher Kjeldsen, Xiaoqin Liu, Kathrine Bang Madsen, Merete Lund Mægbæk, Trine Munk‐Olsen

**Affiliations:** ^1^ Research Unit of Child and Adolescent Psychiatry, Department of Clinical Research University of Southern Denmark Odense Denmark; ^2^ Clinical Pharmacology, Pharmacy and Environmental Medicine, Department of Public Health University of Southern Denmark Odense Denmark; ^3^ Department of Clinical Research University of Southern Denmark Odense Denmark; ^4^ Department of Gynecology and Obstetrics Odense University Hospital Odense Denmark; ^5^ NCRR‐National Centre for Register‐Based Research, Department of Public Health Aarhus University Aarhus Denmark

**Keywords:** cohort studies, concordance, measurement, postpartum depression

## Abstract

**Introduction:**

Postpartum depression (PPD) can be measured in various ways, including questionnaire‐based symptom scores or through administrative health databases. We examined the overlap between symptom‐based and register‐based PPD definitions and assessed the consistency in exposure‐outcome associations.

**Methods:**

We linked Danish nationwide health registers to PPD screening records (Edinburgh Postnatal Depression Scale, EPDS) at 2 months postpartum from women screened between January 1, 2015, and December 31, 2021. We defined symptom‐based PPD as an EPDS ≥ 11, and register‐based PPD as an antidepressant prescription or hospital depression diagnosis within 1 year postpartum. We estimated the overlap between women with register‐based and symptom‐based PPD, as well as between continuous EPDS scores and register‐based treatment indicators. We evaluated consistency between the two definitions using logistic regression analyses for socioeconomic, obstetric, demographic‐ and health‐related exposures from which we obtained odds ratios (ORs) for each PPD definition and calculated ratio of odds ratios (ROR).

**Results:**

Among 157,193 mothers (132,593 unique), 11,193 (7.1%) had symptom‐based PPD and 2409 (1.5%) had register‐based PPD. Of those with symptom‐based PPD, 8.8% also had register‐based PPD, while 40.8% of those with register‐based PPD had symptom‐based PPD. We observed a higher overlap with higher EPDS scores. Consistency was highest for obstetric variables but varying for demographic‐ and health‐related exposures (ROR register‐based vs. symptom‐based 1.84 [95% CI 1.67–2.03] for psychiatric history and 0.75 [95% CI 0.68–0.83] for primiparity).

**Conclusion:**

We observed substantial differences and limited overlap between symptom‐based and register‐based PPD. Register‐based measures capture hospital‐based diagnoses and pharmacological treatment only, but not all treatment within the healthcare system, and differences may reflect a combination of severity, symptom transience, misclassification, and timing of measurement. Consistency in exposure‐outcome associations varied, particularly for certain exposures. Our findings underline the importance of considering both the nature and implications of PPD definitions in epidemiological research.

## Keypoints

- Significant outcomes
  ○ Symptom‐based postpartum depression (PPD) yielded markedly higher prevalence estimates (7.1%) compared to register‐based PPD (1.5%).
  ○ There was limited overlap between the two definitions: Only 8.8% with symptom‐based PPD also had register‐based PPD, while 40.8% with register‐based PPD also had symptom‐based PPD.
  ○ We found only moderate agreement in exposure–outcome associations when comparing the two definitions of PPD.
- Limitations
  ○ Register‐based PPD did not capture women diagnosed in general practice with no pharmacological treatment or those treated solely in municipality‐based services.
  ○ Symptom‐based PPD was assessed only once, approximately 2 months postpartum, and therefore does not capture symptoms occurring outside this time point.

## 1 Background

Postpartum depression (PPD) is a common mental disorder following childbirth, with an estimated prevalence of 10%–15% among new mothers [1, 2]. Postpartum depression can have serious consequences, including impaired mother‐baby bonding, risk of subsequent maternal depressive episodes, difficulties in partner relationships, and a negative impact on infant cognitive and language development [3].

In clinical settings, PPD symptoms are often identified using the Edinburgh Postnatal Depression Scale (EPDS), a questionnaire developed for measuring depressive and anxiety symptoms specifically adapted to the postpartum period [4, 5]. Based on the EPDS screening result, women can be referred for further assessment or treatment. Treatment for PPD may include individual or group therapy, or in more severe cases, pharmacological treatment or referral to specialized psychiatric care [2].

The EPDS is widely used in clinical and epidemiological research to measure PPD symptoms, often by applying a cut‐off score to define case status [6]. A limitation of using the EPDS for epidemiological research is that the collection of such data can be financially and logistically resource intensive. An alternative approach when studying PPD is to use available information from administrative health data, such as population‐wide registers. While administrative databases rarely capture all treatment pathways for PPD, cases may be identified from records of a depression diagnosis in the postpartum period. In settings where depression diagnoses are only recorded at specialized psychiatric care facilities, register‐based PPD may be supplemented by redeemed antidepressant prescriptions as a marker of depression, as this may also capture some women with PPD treated in primary care [7, 8, 9].

Defining PPD using the EPDS reflects the presence of depressive symptoms, whereas defining PPD from registers identifies women with recorded information on treatment for PPD, which may reflect only a subset of treated cases, particularly those with more severe depression. Since not all women whose EPDS scores indicate PPD will require or receive treatment as recorded in health registers, some disagreement between the two definitions is therefore expected. In particular, milder cases of PPD that are treated non‐pharmacologically in primary care may not appear in health registers. However, the extent of overlap between women identified with symptom‐based and register‐based PPD is not well described. Further, it remains uncertain whether the two measures capture comparable populations when used in epidemiological research, or whether misclassification of case status may lead to inconsistencies in exposure‐outcome associations.

Prior research has indicated limited overlap between survey‐based and register‐based measures of depression, with one Danish study reporting limited overlap between an EPDS ≥ 13 and antidepressant use; however, with a similar risk factor profile between the two measures [10]. Another study has reported poor agreement between self‐reported and register‐based measures of depression outside the perinatal period [11].

Investigating the overlap between symptom‐based and register‐based measures of PPD provides insights into how PPD is detected and treated as reflected in administrative data, as well as how and to what extent it is captured across different measurement methods. Additionally, it informs how the choice of outcome definition may influence findings in epidemiological research, providing insights into future studies in the PPD research field.

## 2 Aim

The aim of the study was to examine the overlap between symptom‐based and register‐based operational definitions of PPD and describe the characteristics of women identified with different combinations of these measures. We further assessed the consistency in exposure‐outcome associations across the two definitions.

## 3 Methods

We conducted a register‐based study linking Danish health registers to screening records of PPD measured using the EPDS among mothers who were screened between 2015 and 2021.

### 3.1 Data Sources

The data platform for this study included the HOPE cohort and Danish nationwide health registers. The HOPE cohort is a representative Danish nationwide cohort containing information on PPD screenings using the EPDS from 170,218 mothers (142,795 unique women) who were screened January 1, 2015, and December 31, 2021 [12]. Note, in this and the following, we consequently referred to birthing individuals as *mothers*, regardless of gender identity. Screening records were collected from health nurses' routine home visits and were available from 67 out of 98 Danish municipalities. Screening records were collected no later than 3 months postpartum (on average 8 weeks postpartum).

Using the unique personal identifier assigned to all individuals residing in Denmark, linkage with nationwide health registers was possible. The Danish National Patient Register (Patient Register) holds information on all in‐ and outpatient hospital diagnoses including specialized psychiatric care since 1995 using the 10th International Classification of Diseases (ICD‐10) classification system [13]. The Danish National Prescription Register (Prescription register) contains information on all redeemed prescriptions at Danish community pharmacies including the date of filling since 1995. Drugs in this register are classified according to the World Health Organization (WHO) Anatomical Therapeutic Chemical (ATC) Classification [14]. The Danish National Health Service Register contains information on health services provided, but not clinical information, at primary care facilities since 1990 [15]. The Danish Medical Birth Register (Birth Register) contains information related to pregnancy and birth since 1973 [16], and Statistics Denmark's socioeconomic registers contain information on sociodemographic factors including education and income level.

### 3.2 Study Population

We included women who were screened between one and 3 months postpartum (~98%) in the HOPE cohort. We applied a two‐year wash‐out period, excluding women who had a history of depression defined as a depression diagnosis (ICD‐10 F32‐33) or a redeemed antidepressant prescription (ATC N06A) 2 years prior to childbirth. This was done in accordance with register‐based studies, defining PPD as a new‐onset depressive episode [7, 17].

### 3.3 Measures of Postpartum Depression

Symptom‐based PPD, defined from questionnaire data, was available through EPDS screening records. The EPDS is a validated and widely used tool to measure depressive and anxiety symptoms in the postpartum period. It consists of 10 questions, each scored from 0 to 3 points, with a total score range of 0 to 30. The EPDS has been validated in a Danish context against the ICD‐10 classification of a depression diagnosis, in which a cut‐off of 11 or more was found to indicate PPD [18]. We included the EPDS as both a continuous variable and a cut‐off ≥ 11 to indicate PPD.

Register‐based PPD was defined using Danish health registers, based on either a primary or secondary depression diagnosis given at a psychiatric hospital facility (ICD‐10: F32‐33) from the Patient Register and/or a redeemed antidepressant prescription (ATC: N06A) from the Prescription Register within 12 months postpartum.

### 3.4 Other Indications of Psychiatric Conditions

To further describe the overlap between PPD symptoms and register‐based treatment indicators, we defined the following indications of conditions within the first year postpartum: (a) Hospital visits at any specialized psychiatric care unit with a psychiatric diagnosis (ICD‐10: F00‐99) defined from the Patient Register, (b) any redeemed psychotropic medication (ATC: N05‐06) defined from the Prescription Register, and (c) psychologist visits referred from GP, identified from the Health Service Register.

### 3.5 Additional Study Measures

We included data on the following variables for descriptive purposes and to evaluate consistency: Maternal age at delivery, parity, multiple births, preterm birth (before 37 weeks of gestation), cesarean section (ICD‐10: O82, O84.2, O84.3, O84.3D or procedure code KMCA10A or KMCA10E), preeclampsia/eclampsia (ICD‐10: O14, O11, O15), gestational diabetes mellitus (GDM) (ICD‐10: O24.4), hyperemesis gravidarum (ICD‐10: O21), postpartum hemorrhage > 500 mL (ICD‐10: O72.0), and admission to neonatal intensive care unit (NICU), all derived from the Birth Register. Psychiatric history, defined as any psychiatric diagnosis (ICD‐10: F00‐F99) or redeemed psychotropic medication (ATC: N05‐06) prior to childbirth, was obtained from the Patient Register and the Prescription Register. Information on highest educational attainment (primary, high school or vocational, short‐ or medium‐cycle, and long‐cycle or PhD) and household income (quintiles in the year preceding childbirth) was collected. Cohabitation status (cohabiting or non‐cohabiting) was determined in the year before childbirth, while ethnicity (Danish or foreign) was classified according to country of origin.

### 3.6 Statistical Analysis

First, to describe the overlap between PPD definitions, we identified the total number of women having an EPDS score ≥ 11, antidepressant prescriptions, depression diagnoses, and the independent agreement with each of the other measures. Additionally, we estimated the conditional probability of being a case within each of the other PPD measures. For a sensitivity analysis, we repeated this using a cut‐off of EPDS ≥ 15 as the PPD definition, which has been suggested as a cut‐off disregarding transient PPD symptoms [19], and restricting the register definition of PPD to only 6 months postpartum. Additionally, we applied a sensitivity analysis assessing the overlap without the application of a washout period and assessing the overlap with anxiety disorders (ICD‐10 F40‐48) and anxiolytics (ATC N05B).

Second, we described pregnancy, birth, and sociodemographic characteristics across groups defined by different combinations of symptom‐based and register‐based PPD. Third, we described the overlap between the continuous EPDS scores and selected treatment indicators including depression diagnosis, antidepressant prescription, psychiatric hospital contacts, psychotropic medication, and referrals to psychiatrists or psychologists as defined above. Within each treatment indicator, we estimated the proportion of individuals receiving treatment within each total EPDS score.

Finally, we assessed the consistency in exposure‐outcome associations across symptom‐based (EPDS‐scores) and register‐based (depression diagnoses or antidepressant prescriptions) definitions of PPD. We performed logistic regression analyses using obstetric, socioeconomic, and demographic‐ and health‐related factors (as defined above) as exposures, estimated separately for each outcome definition. Continuous and categorical exposures were dichotomized as low maternal age (< 25 years), low education (primary education), and low income (lowest income quintile). All models were adjusted for age, parity, income, education, country of origin, cohabitation status, and psychiatric history, except when the exposure of interest overlapped with these variables. For socioeconomics, income was not adjusted when education was the exposure and vice versa. For each exposure, we estimated odds ratios (ORs) for both outcomes and calculated the ratio of odds ratios (ROR) (register‐based vs. symptom‐based). We obtained 95% confidence intervals (CIs) for the RORs using a parametric bootstrap with 1000 replications, using unique mothers as the sampling unit to account for mothers contributing with more than one birth.

All analyses were conducted using R Studio version 4.2.

### 3.7 Ethics

The study was approved by the Danish Data Protection Agency through institutional registration at Aarhus University (journal no. 2016‐051‐000001, serial no. 2304). In accordance with Danish law, ethical approval and informed consent are not required for register‐based research. Access to EPDS questionnaire data from medical records was granted by the Danish Patient Safety Authority, and the Danish Data Protection Agency approved linkage of these data with national registers via Statistics Denmark.

## 4 Results

**FIGURE 1:**
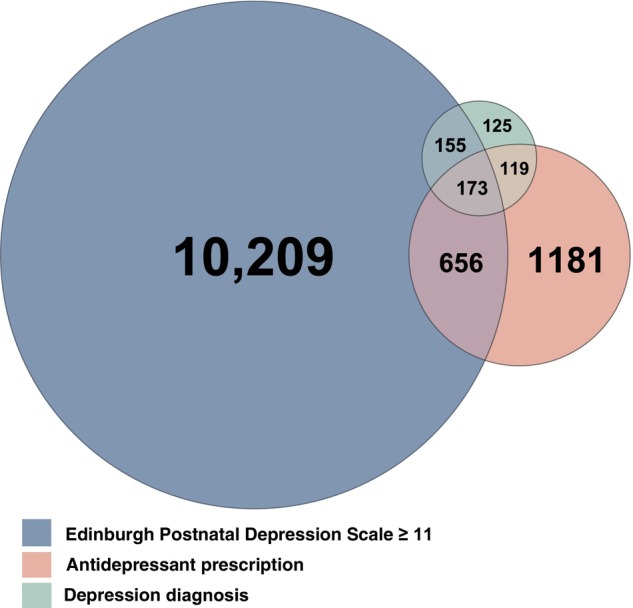
Number of women identified with postpartum depression defined using the Edinburgh Postnatal Depression Scale, antidepressant prescriptions, and depression diagnoses. Circle sizes and overlaps are scaled to reflect the relative proportions.

**FIGURE 2:**
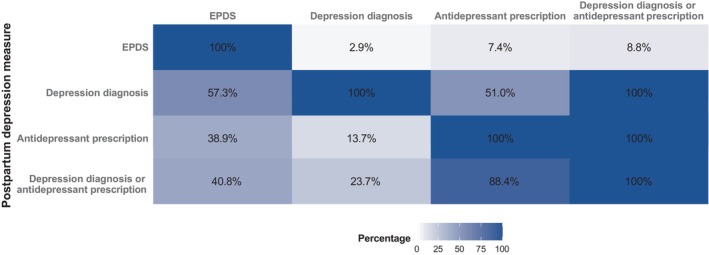
Conditional probabilities of overlap between different postpartum depression measures. Among women identified by the measure in each row, the values show the probability of also being identified by the measure in the corresponding column.

The study population included births from 157,193 mothers (132,493 unique) with no record of depression 2 years prior to childbirth (Figure S1). Of these, 12,618 (8.0%) had either an EPDS ≥ 11, antidepressant prescription, or depression diagnosis postpartum. Specifically, 11,193 (7.1%) had an EPDS ≥ 11, 2129 (1.4%) had an antidepressant prescription, and 572 (0.4%) had a depression diagnosis, while 2409 (1.5%) had either a prescription or diagnosis (Figure 1). Among women with an EPDS ≥ 11, 2.9% also had a depression diagnosis, 7.4% had an antidepressant prescription, and 9.0% had either of the two (Figure 2). Among women with a depression diagnosis, 57.3% had an EPDS ≥ 11, and 51.0% had an antidepressant prescription. Among women with an antidepressant prescription, 38.9% had an EPDS ≥ 11, and 13.7% had a depression diagnosis.

Compared to women with neither symptom‐based (EPDS ≥ 11) nor register‐based (prescription or diagnosis) PPD, women with one or both were more likely to be non‐cohabiting, have low education and income, have a psychiatric history, and to have birth‐ and obstetrical complications (Table 1). Among women with any indication of PPD, those with register‐based PPD only were the most socially disadvantaged, particularly in terms of education and income, followed by women with both symptom‐based and register‐based PPD, and least so among those with symptom‐based PPD only. Women with register‐based PPD also had a higher proportion of psychiatric history (53.1% for register‐based only and 51.2% for those with both) compared to those with no PPD (21.1%) or symptom‐based PPD only (32.8%) (Table 1).

**TABLE 1:** Descriptive characteristics of the study population according to postpartum depression identified by symptom‐based (EPDS ≥ 11) and register‐based (antidepressant prescriptions or depression diagnoses) measures and their overlap.

	No symptom‐based or register‐based PPD	Symptom‐based PPD only	Register‐based PPD only	Both symptom and register‐based PPD
*n*	144,575	10,209	1425	984
EPDS score, median (IQR)	4 (2–6)	13 (11–15)	6 (4–8)	15 (12–18)
Weeks from birth to EPDS screening, median (IQR)	8 (8–9)	8 (8–9)	8 (8–9)	8 (8–9)
Weeks from birth to register‐based PPD, median (IQR)	—	—	32 (19–43)	19 (10–34)
Age (median [IQR])	30 (27–33)	30 (27–33)	29 (26–33)	30 (26–33)
Cohabitation status, *n* (%)				
Cohabiting	133,813 (92.6)	9240 (90.5)	1267 (88.9)	881 (89.5)
Non‐cohabiting	9442 (6.5)	877 (8.6)	143 (10.0)	94 (9.6)
Educational level, *n* (%)				
Primary	12,659 (8.8)	1124 (11.0)	271 (19.0)	157 (16.0)
High school or vocational	42,130 (29.1)	3026 (29.6)	520 (36.5)	371 (37.7)
Short‐ or medium cycle	53,202 (36.8)	3643 (35.7)	423 (29.7)	276 (28.0)
Long‐cycle or PhD	35,757 (24.7)	2314 (22.7)	199 (14.0)	174 (17.7)
Income quintile, *n* (%)				
Lowest	28,060 (19.4)	2480 (24.3)	438 (30.7)	291 (29.6)
Second	28,603 (19.8)	2130 (20.9)	320 (22.5)	215 (21.8)
Third	28,826 (19.9)	1967 (19.3)	287 (20.1)	189 (19.2)
Fourth	29,082 (20.1)	1833 (18.0)	205 (14.4)	148 (15.0)
Highest	29,224 (20.2)	1741 (17.1)	170 (11.9)	134 (13.6)
Danish country of origin, *n* (%)	124,173 (85.9)	8375 (82.0)	1258 (88.2)	855 (87.9)
Psychiatric history, *n* (%)	30,604 (21.2)	3347 (32.8)	757 (53.1)	504 (51.2)
Primiparous, *n* (%)				
Primiparous	76,401 (52.8)	5702 (55.9)	666 (46.7)	516 (52.4)
Multiparous	67,047 (46.4)	4415 (43.2)	747 (52.4)	461 (46.8)
Preeclampsia/Eclampsia, *n* (%)	4818 (3.3)	404 (4.0)	41 (2.9)	53 (5.4)
Gestational diabetes, *n* (%)	6712 (4.6)	564 (5.5)	82 (5.8)	66 (6.7)
Hyperemesis gravidarum, *n* (%)	3405 (2.4)	342 (3.3)	58 (4.1)	51 (5.2)
Postpartum hemorrhage, *n* (%)	10,827 (7.5)	800 (7.8)	122 (8.6)	88 (8.9)
Preterm birth, *n* (%)	5665 (3.9)	513 (5.0)	67 (4.7)	61 (6.2)
C‐section, *n* (%)	20,365 (14.1)	1685 (16.5)	239 (16.8)	205 (20.8)
Neonatal intensive care admission, *n* (%)	11,323 (7.8)	987 (9.7)	123 (8.6)	124 (12.6)

*Note:* Some mutually exclusive characteristics do not sum up to 100% because of missing data.

**FIGURE 3:**
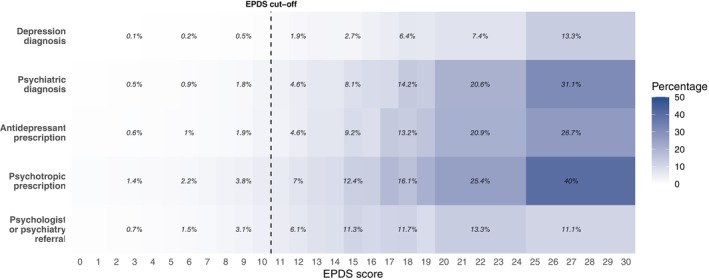
Proportion of register‐based depression and psychiatric treatment outcomes by total EPDS score. EPDS scores 0–1, 20–24, and 25–30 are collapsed due to limited number of cases.

Figure 3 describes the overlap between total EPDS score and depression and psychiatric treatment outcomes. For all treatment outcomes, we observed an increasing overlap with higher total EPDS score. Among women with an EPDS score of 25–30, the overlap was highest for any psychotropic prescription (40%), followed by any psychiatric diagnosis (31.1%), antidepressant prescription (26.7%), depression diagnosis (13.3%), and psychologist or psychiatry referrals from general practice (11.1%) (Figure 3).

**FIGURE 4:**
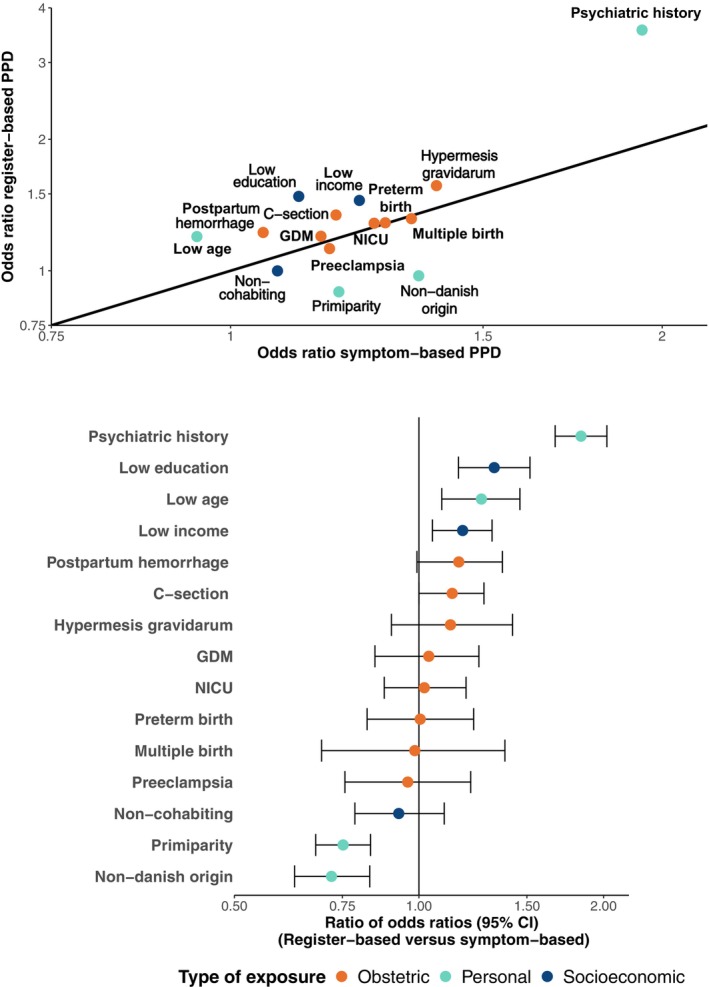
Odds ratios (upper panel) and ratio of odds ratios (lower panel) of register‐ and symptom‐based postpartum depression (PPD) measures across obstetric, demographic and health‐related, and socioeconomic exposures. All analyses are adjusted for age, parity, income, education, country of origin, cohabitation status, and psychiatric history except when the variable is the exposure of interest. The solid line (upper panel) and a ROR of 1 (lower panel) reflect a high degree of consistency between the two outcome definitions. Across analyses, a maximum of 4107 individuals (2.6%) were excluded due to missing data.

Figure 4 contains ORs and ROR for exposure‐outcome associations with register‐based and symptom‐based PPD. For socioeconomic exposures, the OR was more elevated for register‐based PPD than symptom‐based PPD with 1.48 vs. 1.12 for low education (ROR 1.33, 95% CI 1.16–1.51) and 1.45 vs. 1.23 for low income (ROR 1.18, 95% CI 1.05–1.32). For non‐cohabitation, ORs were more similar with 1.00 for register‐based PPD and 1.08 for symptom‐based PPD (ROR 0.93, 95 CI 0.79–1.10). Among demographic and health‐related exposures, OR of register‐based PPD was more elevated than symptom‐based for psychiatric history with 3.56 vs. 1.94 (ROR 1.84, 95% CI 1.67–2.03) and low age with 1.10 vs. 0.95 (ROR 1.26, 95% CI 1.09–1.46). The OR was less elevated for register‐based PPD than symptom‐based PPD for primiparity with 0.89 vs. 1.19 (ROR 0.75, 95% CI 0.68–0.83) and non‐Danish origin with 0.97 vs. 1.35 (ROR 0.72, 95% CI 0.63–0.83). ORs for obstetric variables were rather similar for symptom‐ and register based PPD, with ROR ranging from 0.96 (95% CI 0.76–1.21) for preeclampsia to 1.16 (95% CI 0.99–1.37) for postpartum hemorrhage (Figure 4, Table S1).

Sensitivity analyses restricting symptom‐based PPD to EPDS **≥**15 increased the overlap with register‐based PPD 15% (Figures S2 and S3), whereas restricting register‐based PPD to a duration of 6 months after childbirth increased the overlap with a symptom‐based PPD to 68% for diagnoses, 52% for prescriptions (Figures S4 and S5). When not applying a washout period, 15% of women with symptom‐based PPD had register‐based PPD (Figure S6). Among women with an EPDS ≥ 11, 5% had an anxiety diagnosis or anxiolytic medication use (Figure S7).

## 5 Discussion

### 5.1 Main Findings

We observed the highest proportion of PPD cases with symptom‐based PPD at 7.1%, whereas register‐based PPD identified markedly fewer cases, at only 1.5%. Only 8.8% of women with symptom‐based PPD also had register‐based PPD, and less than half of the women with register‐based PPD had symptom‐based PPD, suggesting a limited overlap between the two case definitions. Importantly, this limited overlap reflects differences between the two operational definitions and does not represent overlap between depressive symptoms and treatment across the healthcare system. We observed a dose–response pattern, where higher EPDS scores were associated with a greater proportion of register‐based cases. However, even among women with the highest EPDS scores (EPDS > 25), less than half were identified in the registers. Additionally, we observed moderate consistency in exposure‐outcome associations using the two PPD outcome definitions. While the direction of associations was generally similar, there was variation in the magnitude of estimated ORs, most pronounced for demographic and health‐related, but also socioeconomic exposures.

### 5.2 Interpretation

Our findings reveal two key observations regarding the identification of PPD: First, symptom‐based and register‐based case definitions yielded markedly different prevalence estimates. Symptom‐based PPD was detected through routine screenings, while register‐based PPD captured only women who sought care and either received pharmacological treatment, a specialist diagnosis, or both. Consequently, the register‐based definition likely reflects moderate to severe cases, providing a conservative estimate that may account for the substantial discrepancy in prevalence.

Second, there was limited overlap between the cases identified by each method. Even though agreement with register‐based indicators increased with higher EPDS scores, many women still had no register‐based diagnosis. The very low overlap observed even among women with high EPDS scores (EPDS > 25) may warrant consideration of how severe symptoms translate into appropriate treatment and care. While the EPDS is validated for detecting PPD, its positive and negative predictive values in the Danish validation are estimated at 49.0% and 98.5%, respectively [18], indicating some degree of misclassification of case status, potentially limiting agreement between measures. Additionally, studies have suggested that the EPDS may capture a high proportion of women with transient depressive symptoms [19]. Thus, even if symptoms meet diagnostic criteria at a specific point in time, women may not seek or receive care if symptoms resolve on their own. Therefore, symptom‐based PPD may capture a broader range of cases, including those not treated or treated outside specialized care, but also some that may not be clinically detectable. Additionally, 5% of mothers with EPDS ≥ 11 had an anxiety diagnosis or redeemed anxiolytics, suggesting that part of the limited overlap may reflect that the EPDS also captures anxiety symptoms.

Additionally, more than half of women with register‐based PPD did not have symptom‐based PPD. While symptom‐based PPD was measured on average 2 months postpartum, register‐based PPD included any prescriptions or diagnoses within the first year postpartum. If some women only developed clinically relevant symptoms after the time of screening, they may not have been captured by the EPDS and only appear in registers. This is supported by the fact that women with only register‐based PPD had a later median time of diagnosis (32 weeks postpartum) compared to those with both symptom‐ and register‐based PPD (19 weeks postpartum). Further, sensitivity analyses restricting to register‐based PPD within 6 months postpartum increased the overlap with symptom‐based PPD, supporting that overlap is greater when timely closer to each other. However, for register‐based PPD, some temporal delay between symptom onset and registration is expected, as symptoms precede diagnosis or treatment recorded in registers, which may account for part of the observed difference in timing between the two measures. Altogether, the limited overlap between the two PPD measurements may reflect both differences in case severity, time window of assessment, and the sensitivity and specificity of the EPDS questionnaire.

We observed only moderate consistency in exposure‐outcome associations using the two case definitions. Notably, the demographic and health‐related exposures showed the lowest consistency across PPD definitions, with some associations differing in direction. Variation in these effect estimates may reflect differences in both symptom severity, recognition, referral, or health‐seeking behaviors. For instance, transient symptoms are more common among primiparous women [19], and thus more likely to be associated with symptom‐based PPD than register‐based PPD. Also, women of non‐Danish origin and non‐cohabiting women had lower risk of register‐based PPD than symptom‐based PPD, which could reflect barriers to access or different help‐seeking behaviors. In contrast, women with a psychiatric history may be more likely to be identified and referred to psychiatric care, which could explain the substantially elevated estimates for register‐based PPD compared to symptom‐based PPD. Thus, differences in observed exposure‐outcome associations may reflect factors influencing the likelihood of being identified with symptom‐based and register‐based PPD. As these factors may also relate specifically to the exposure of interest, the implications of the PPD definition used must be carefully considered in epidemiological research.

### 5.3 Comparison With Previous Studies

Holm et al. described quantitative comparisons between EPDS scores and register‐based treatment indicators, but applied slightly different definitions [10]. They found that only 4.5% of women with EPDS ≥ 13 had an antidepressant prescription or depression diagnosis within 6 months, and that only 44% of women with a prescription or diagnosis had an EPDS ≥ 13. Although consistent with our finding of limited overlap between the two measures, they concluded that the two measures shared a similar risk factor profile. However, this was based on a pooled analysis of multiple exposures, which may obscure the variation in how specific exposures relate to the likelihood of being identified with symptom‐based versus register‐based PPD. While comparisons with studies outside Denmark are limited due to differences in healthcare organization and data availability, findings from Sweden similarly suggest that a substantial proportion of women with positive EPDS scores are not identified in the healthcare system [20].

Weye et al. compared the overlap between symptom‐based depression using the Major Depression Inventory and antidepressant prescriptions or depression diagnoses in a general population (i.e., outside the perinatal period) [11]. They found a higher overlap, namely that among individuals with symptom‐based depression, 51% had a prescription or diagnosis during a 12‐year period. While this could reflect that depressive symptoms are less transient outside the perinatal period, it is also possible that the higher overlap is due to the 12‐year period to assess register‐based depression, where a longer period would increase the likelihood of an overlap.

### 5.4 Strengths and Limitations

A key strength of this study included the use of the HOPE cohort, a large sample of new mothers with low risk of selection bias [12]. The linkage of screening records to register‐based information on hospital‐based psychiatric diagnoses and redeemed prescriptions enabled a unique comparison of the two measures. However, the following limitations should be acknowledged: We did not have information on women who received for example, therapy treatment in municipality‐based care or non‐pharmacological treatment in primary care and therefore did not capture all women who received treatment in our register‐based definitions. Having had this information, we would hypothesize a higher overlap between measures. Second, although generally representative, the HOPE cohort is underrepresented in relation to women of non‐Danish origin, which could have obscured exposure‐outcome associations for this specific variable. For prescriptions, we did not restrict to prescriptions with depression as the treatment indication and we may have captured women treated with antidepressants due to other conditions. However, the indication for depression treatment in the perinatal period has been found to be fairly high [21]. Finally, our comparisons are context‐dependent, and the agreement between symptom‐based measures and administrative health data may vary across settings. The overlap observed in this study may therefore not generalize to other countries or healthcare systems.

## 6 Conclusion

In this study, we observed substantial differences between symptom‐based and register‐based definitions of PPD. Symptom‐based PPD identified markedly more cases than register‐based PPD, and there was a limited overlap between the two measures. While symptom‐based PPD is likely to capture a broader spectrum of severity, register‐based PPD may reflect more severe or persistent cases. The limited overlap may reflect both case severity, sensitivity of the EPDS questionnaire, symptom transience, and substantial differences in the timing of PPD measurement. Additionally, we found only a moderate consistency in exposure‐outcome associations across the two measures, with the strongest agreement for obstetric exposures and greater variation for socioeconomic and demographic and health‐related factors. Jointly, these findings underline the importance of considering both the nature and limitations of different PPD definitions in epidemiological research, as specific exposures may differentially influence the likelihood of being identified across definitions and thus affect observed associations.

## Author Contributions

Conceptualization: S.E., M.B., T.M.‐O. Data access: S.E., X.L. Formal analysis: S.E. Funding acquisition: S.E., T.M.‐O. Writing – original draft: S.E. Writing – review and editing: S.E., M.B., M.‐M.Z.K., K.B.M., M.L.M., X.L., T.M.‐O. Supervision: T.M.‐O., M.B., X.L.

## Funding

The Novo Nordisk Foundation (S.E., M.B., T.M.‐O., grant number NNF21OC0072397), The Psychiatric Research Fund in the Region of Southern Denmark (S.E., grant number A5752), and The Region of Southern Denmark (S.E., grant number A1784). Remaining authors: None.

## Ethics Statement

The study was approved by the Danish Data Protection Agency through institutional registration at Aarhus University (journal no. 2016‐051‐000001, serial no. 2304).

## Consent

In accordance with Danish law, informed consent is not required for register‐based research. Access to EPDS questionnaire data from medical records was granted by the Danish Patient Safety Authority, and the Danish Data Protection Agency approved linkage of these data with national registers via Statistics Denmark.

## Conflicts of Interest

T.M.‐O. has received a speaker honorarium from Lundbeck A/S. Remaining authors: None.

## Supporting information


**Table S1:** Odds ratio and ratio of odds ratios of register‐ and symptom‐based postpartum depression measures across obstetric, personal, and socioeconomic exposures.
**Figure S1:** Flowchart of the study population.
**Figure S2:** Number of women identified with postpartum depression defined using the Edinburgh postnatal depression scale with PPD defined as a score of 15 or more, antidepressant prescriptions and depression diagnoses. Circle sizes and overlaps are scaled to reflect the relative proportions.
**Figure S3:** Conditional probabilities of overlap between different postpartum depression measures, defining symptom‐based PPD as EPDS ≥ 15. Among women identified by the measure in each row, the values show the probability of also being identified by the measure in the corresponding column.
**Figure S4:** Number of women identified with postpartum depression defined using the Edinburgh postnatal depression scale, and antidepressant prescriptions and depression diagnoses within 6 months after childbirth. Circle sizes and overlaps are scaled to reflect the relative proportions.
**Figure S5:** Conditional probabilities of overlap between different postpartum depression measures, defining register‐based PPD within a duration of 6 months after childbirth. Among women identified by the measure in each row, the values show the probability of also being identified by the measure in the corresponding column.
**Figure S6:** Conditional probabilities of overlap between different postpartum depression measures, without application of a two‐year washout period in the study population (Total *n* = 166,645). Among women identified by the measure in each row, the values show the probability of also being identified by the measure in the corresponding column.
**Figure S7:** Conditional probabilities of overlap between EPDS and postpartum anxiety measures. Among women identified by the measure in each row, the values show the probability of also being identified by the measure in the corresponding column.

## Data Availability

The study uses individual‐level register data, which are confidential and cannot be shared according to Danish regulations.
