# Supplementary material for: Postpartum Depression: Overlap Between Symptom‐Based and Register‐Based Measures
Source: Acta Psychiatr Scand. 2026 Apr 14;154(1):50–9. doi: 10.1111/acps.70090 (PMC13242969; doi:10.1111/acps.70090)
Supplement: Supplementary file 1 — Table S1: Odds ratio and ratio of odds ratios of register‐ and symptom‐based postpartum depression measures across obstetric, personal, and socioeconomic exposures. Figure S1: Flowchart of the study population. Figure S2: Number of women identified with postpartum depression defined using the Edinburgh postnatal depression scale with PPD defined as a score of 15 or more, antidepressant prescriptions and depression diagnoses. Circle sizes and overlaps are scaled to reflect the relative proportions. Figure S3: Conditional probabilities of overlap between different postpartum depression measures, defining symptom‐based PPD as EPDS ≥ 15. Among women identified by the measure in each row, the values show the probability of also being identified by the measure in the corresponding column. Figure S4: Number of women identified with postpartum depression defined using the Edinburgh postnatal depression scale, and antidepressant prescriptions and depression diagnoses within 6 months after childbirth. Circle sizes and overlaps are scaled to reflect the relative proportions. Figure S5: Conditional probabilities of overlap between different postpartum depression measures, defining register‐based PPD within a duration of 6 months after childbirth. Among women identified by the measure in each row, the values show the probability of also being identified by the measure in the corresponding column. Figure S6: Conditional probabilities of overlap between different postpartum depression measures, without application of a two‐year washout period in the study population (Total n = 166,645). Among women identified by the measure in each row, the values show the probability of also being identified by the measure in the corresponding column. Figure S7: Conditional probabilities of overlap between EPDS and postpartum anxiety measures. Among women identified by the measure in each row, the values show the probability of also being identified by the measure in the corresponding column. [file ACPS-154-50-s001.docx]

**Supplementary material**

**Table S1: Odds ratio and ratio of odds ratios of register- and symptom-based postpartum depression measures across obstetric, personal, and socioeconomic exposures.**

| **Exposure** | **Total population** | **Number of exposed** | **OR, Register-based PPD** | **OR, Symptom-based PPD** | **ROR (95% CI)** |
| --- | --- | --- | --- | --- | --- |
| **C-section** | 153086 | 22089 | 1.34 | 1.18 | 1.13 (1.00-1.28) |
| **Gestational diabetes** | 153086 | 7284 | 1.20 | 1.16 | 1.04 (0.85-1.25) |
| **Hypermesis gravidarum** | 153086 | 3767 | 1.57 | 1.39 | 1.13 (0.90-1.42) |
| **Low age** | 153086 | 14947 | 1.20 | 0.95 | 1.26 (1.09-1.46) |
| **Low education** | 153791 | 14009 | 1.48 | 1.12 | 1.33 (1.16-1.52) |
| **Low income** | 153815 | 30615 | 1.45 | 1.23 | 1.18 (1.05-1.32) |
| **Multiple birth** | 153086 | 1846 | 1.32 | 1.34 | 0.98 (0.69-1.38) |
| **NICU** | 153086 | 12318 | 1.28 | 1.26 | 1.02 (0.88-1.19) |
| **Non-cohabiting** | 153086 | 10284 | 1.00 | 1.08 | 0.93 (0.79-1.10) |
| **Non-danish origin** | 153086 | 20755 | 0.97 | 1.35 | 0.72 (0.63-0.83) |
| **Postpartum hemorrhage** | 153086 | 11626 | 1.22 | 1.05 | 1.16 (0.99-1.37) |
| **Preeclampsia** | 153086 | 5222 | 1.12 | 1.17 | 0.96 (0.76-1.21) |
| **Preterm birth** | 153086 | 6182 | 1.29 | 1.28 | 1.00 (0.82-1.23) |
| **Primiparity** | 153086 | 81907 | 0.89 | 1.19 | 0.75 (0.68-0.83) |
| **Psychiatric history** | 153086 | 34446 | 3.56 | 1.94 | 1.84 (1.67-2.03) |
| All analyses are adjusted for age, parity, income, education, country of origin, cohabitation status and psychiatric history except when the variable is the exposure of interest. Abbreviations: PPD; Postpartum depression, NICU; Neonatal intensive care unit | | | | | |

**
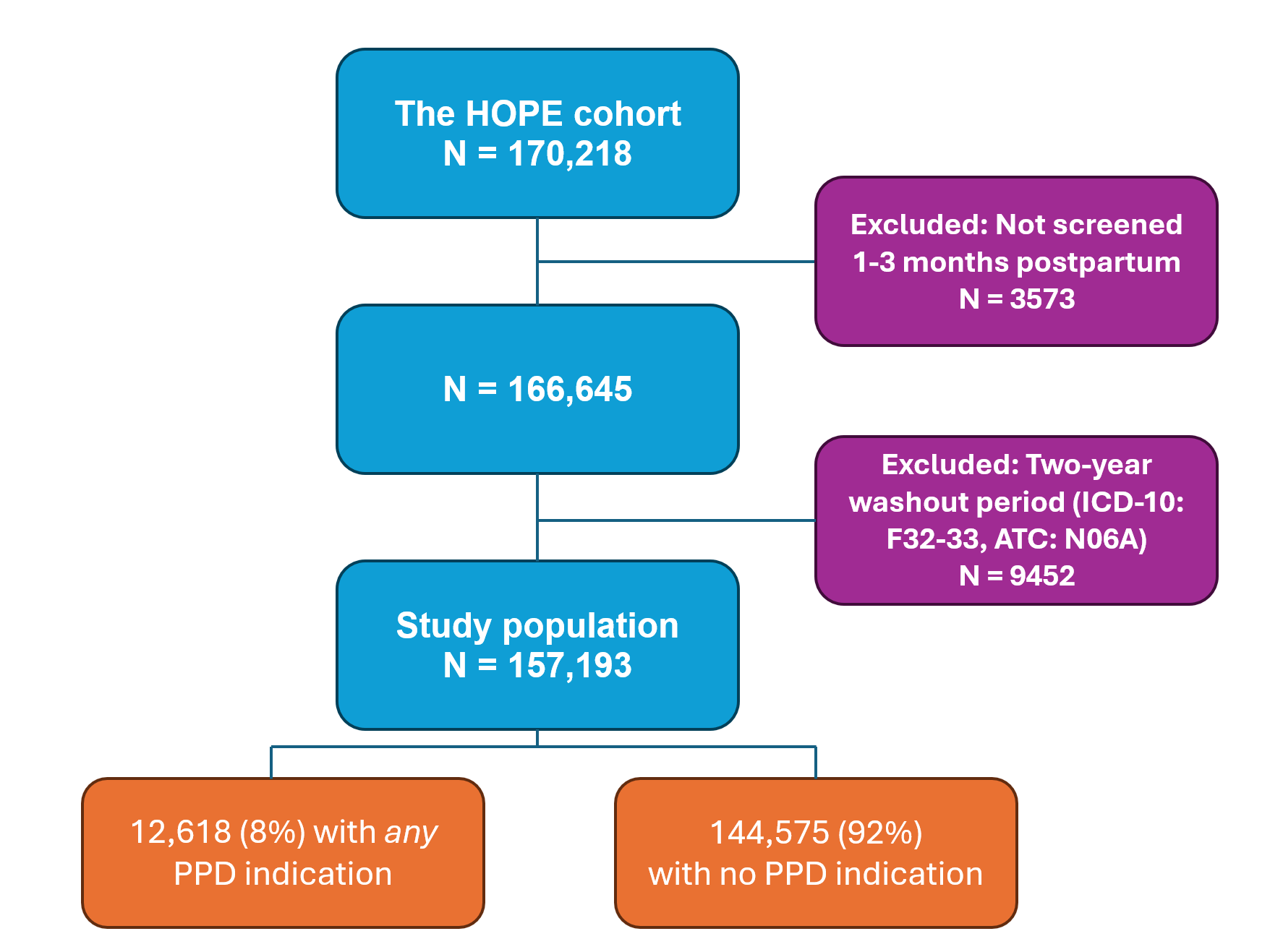
**

**Fig. S1: Flowchart of the study population**

**
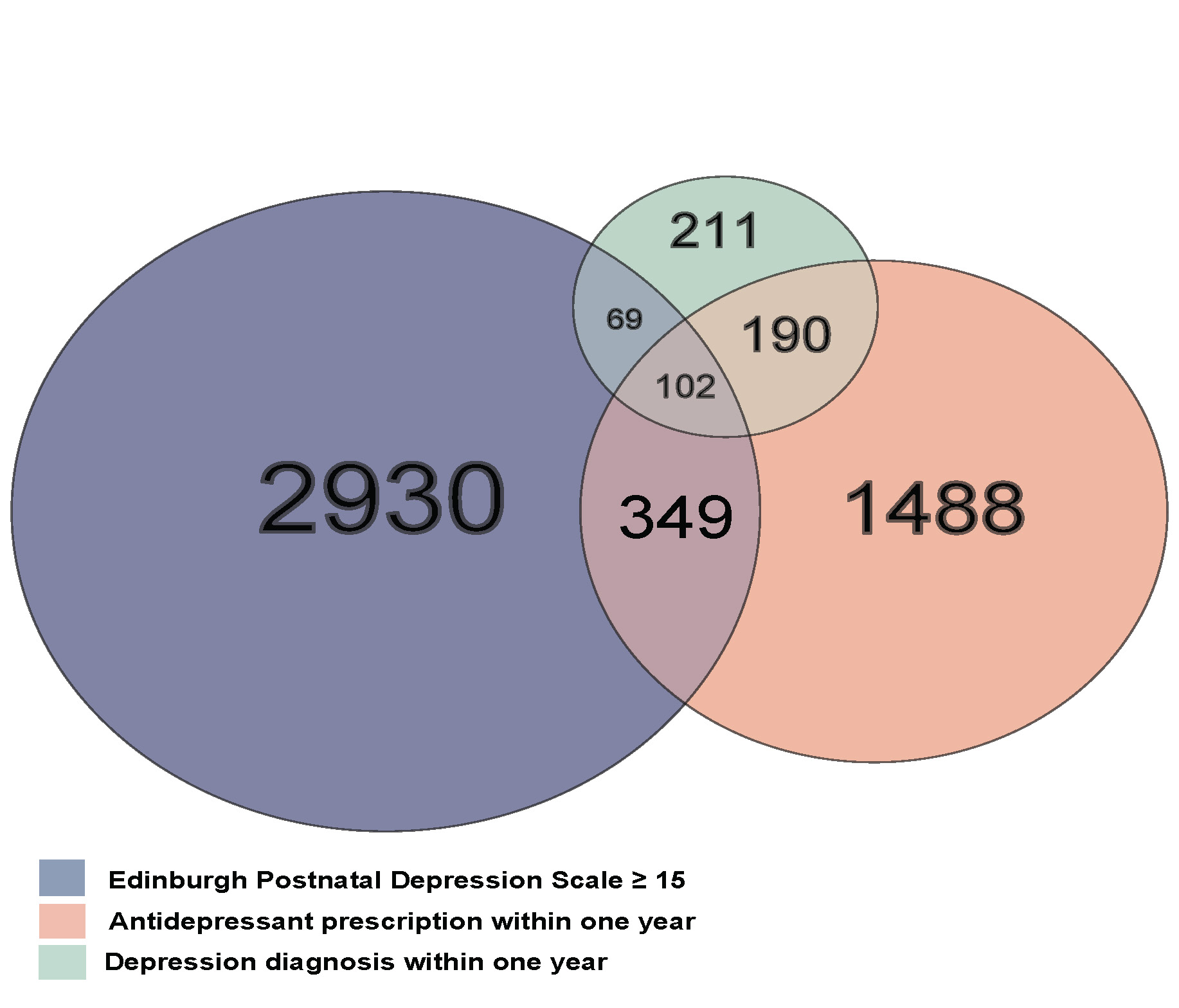
**

**Fig. S2: Number of women identified with postpartum depression defined using the Edinburgh postnatal depression scale with PPD defined as a score of 15 or more, antidepressant prescriptions and depression diagnoses. Circle sizes and overlaps are scaled to reflect the relative proportions.**

**
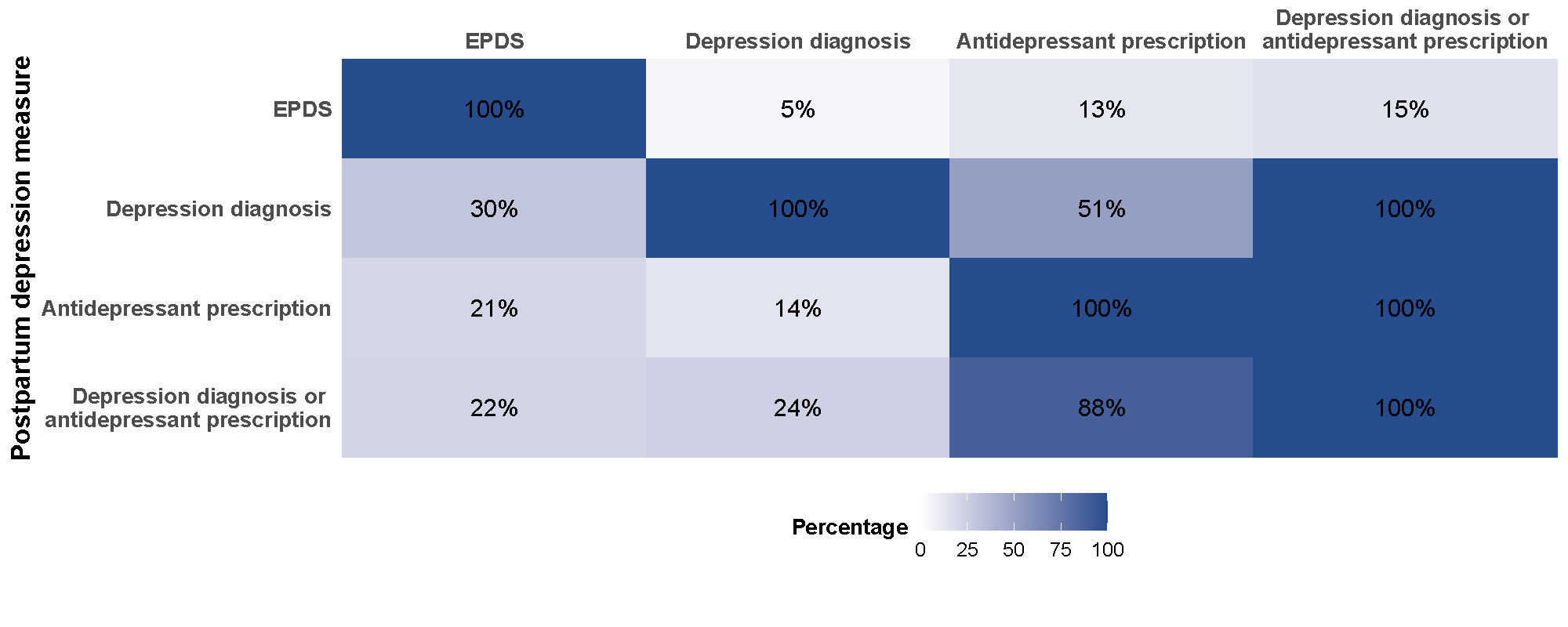
**

**Fig. S3: Conditional probabilities of overlap between different postpartum depression measures, defining symptom-based PPD as EPDS ≥ 15. Among women identified by the measure in each row, the values show the probability of also being identified by the measure in the corresponding column.**

**
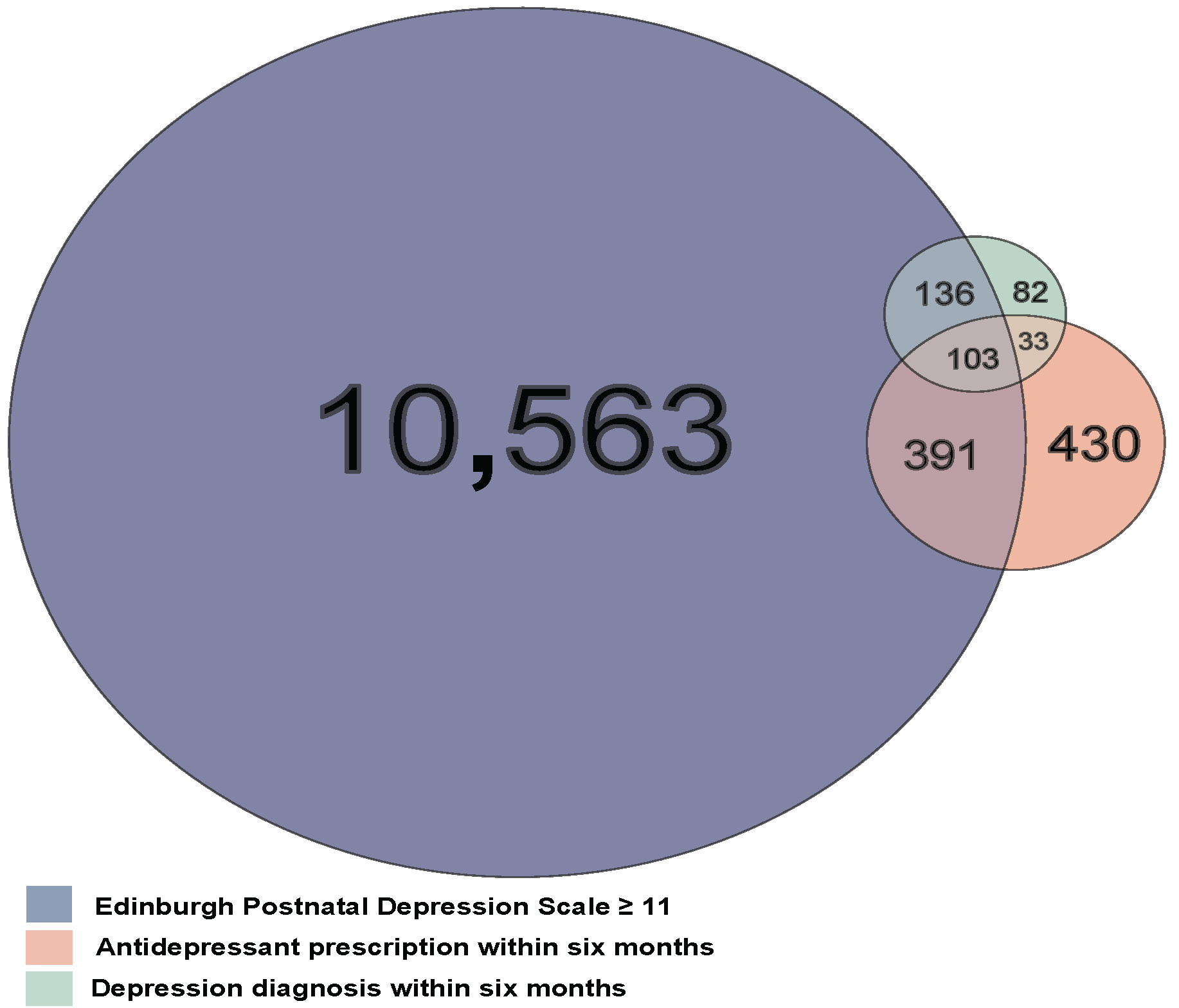
**

**Fig. S4: Number of women identified with postpartum depression defined using the Edinburgh postnatal depression scale, and antidepressant prescriptions and depression diagnoses within six months after childbirth. Circle sizes and overlaps are scaled to reflect the relative proportions.**

**
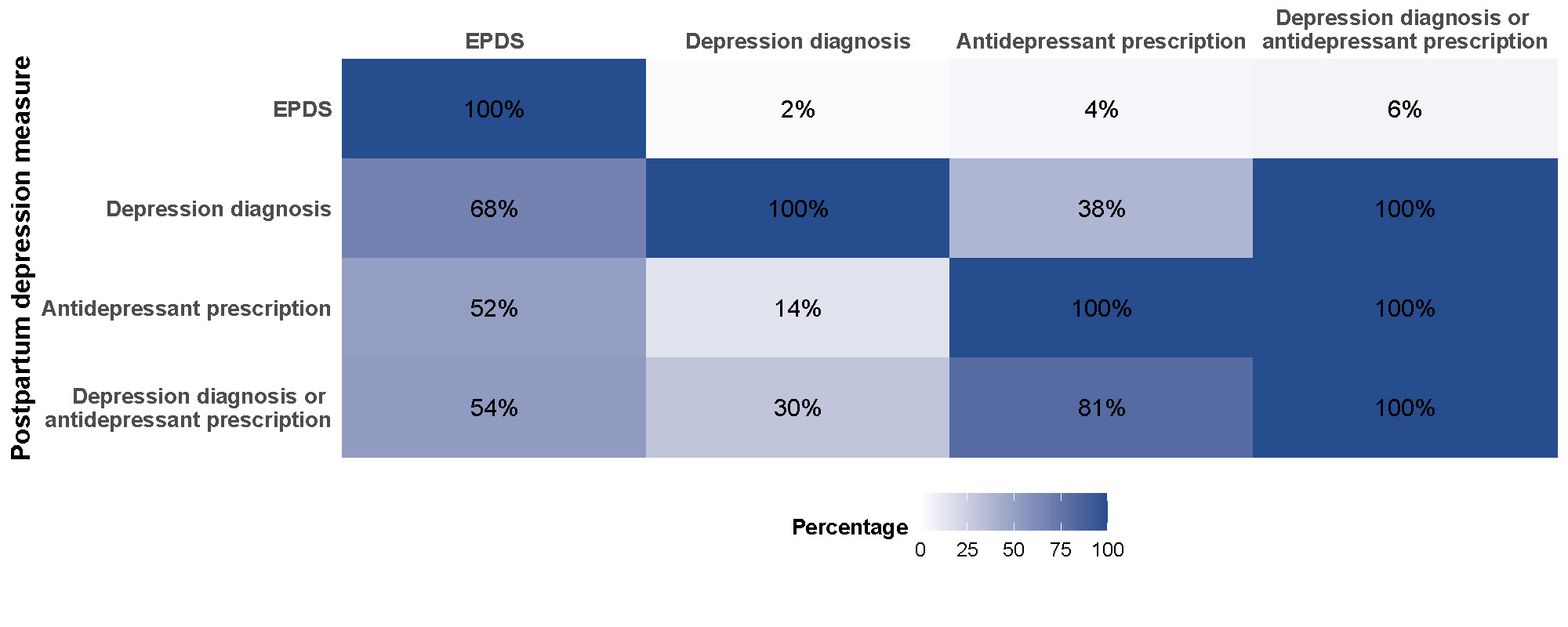
Fig. S5: Conditional probabilities of overlap between different postpartum depression measures, defining register-based PPD within a duration of six months after childbirth. Among women identified by the measure in each row, the values show the probability of also being identified by the measure in the corresponding column.**

**
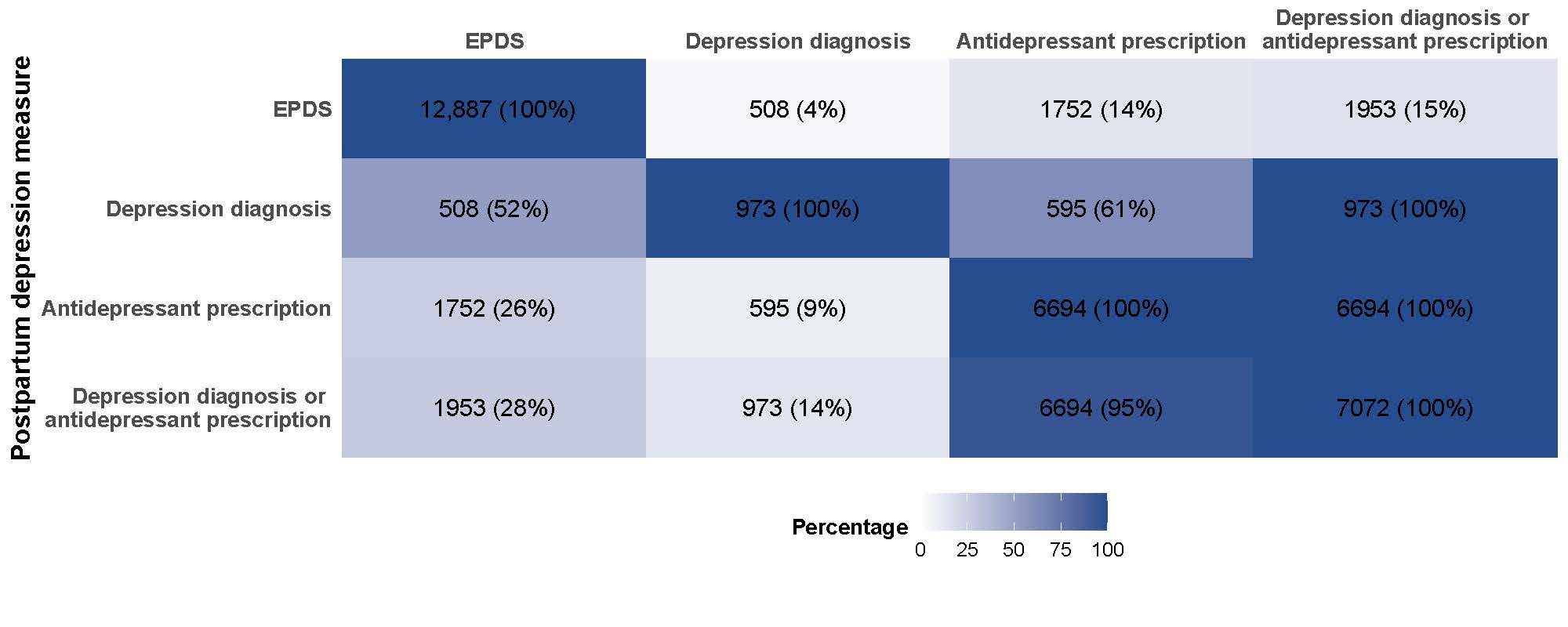
**

**Fig. S6 Conditional probabilities of overlap between different postpartum depression measures, without application of a two-year washout period in the study population (Total n = 166,645). Among women identified by the measure in each row, the values show the probability of also being identified by the measure in the corresponding column**

**
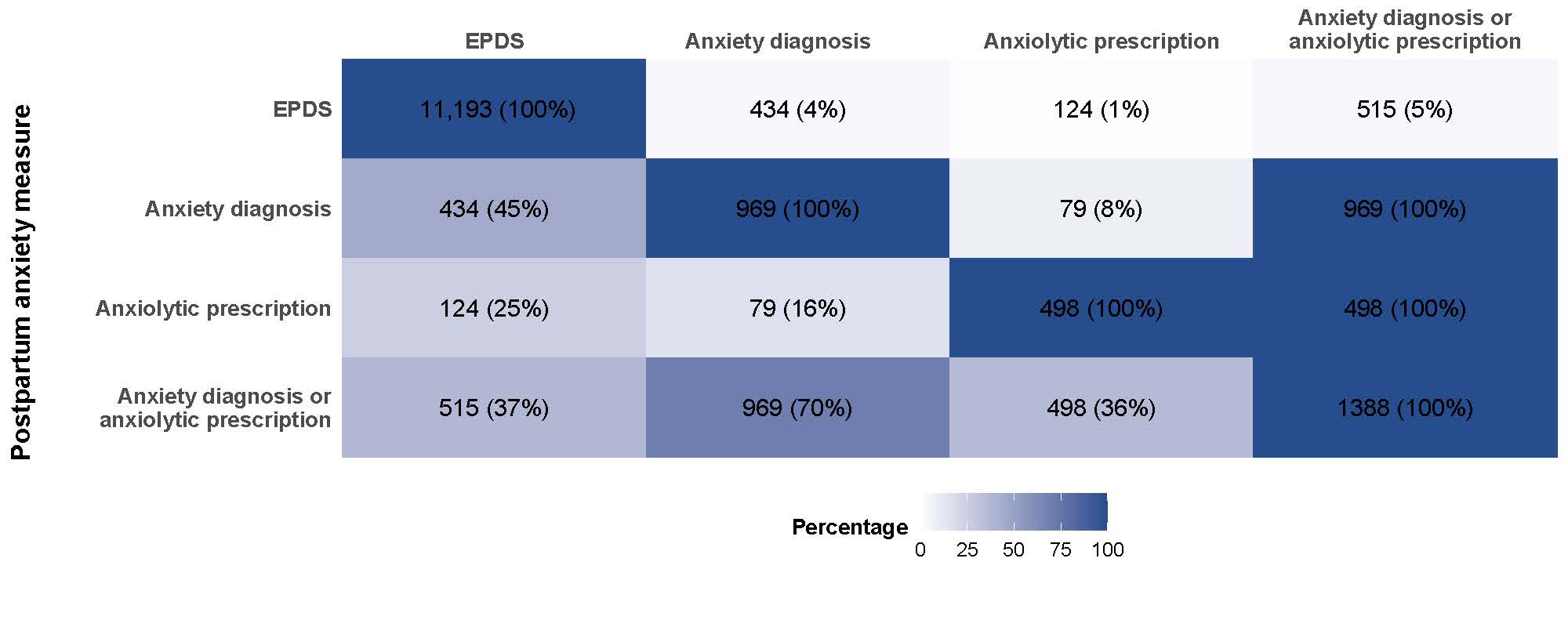
Fig. S7 Conditional probabilities of overlap between EPDS and postpartum anxiety measures. Among women identified by the measure in each row, the values show the probability of also being identified by the measure in the corresponding column**
